# Craving Prediction From fMRI Drug Cue Reactivity in Methamphetamine Use Disorder: A Parsimonious Neurobiological Model

**DOI:** 10.1002/brb3.70991

**Published:** 2025-10-20

**Authors:** Hajar Mahdavi‐Doost, Ghazaleh Soleimani, Kelvin O. Lim, Hamed Ekhtiari

**Affiliations:** ^1^ Department of Psychology University of Minnesota Minneapolis Minnesota USA; ^2^ Department of Psychiatry and Behavioral Sciences University of Minnesota Minneapolis Minnesota USA; ^3^ Laureate Institute for Brain Research Tulsa Oklahoma USA

**Keywords:** addiction, biomarker, Craving, fMRI, machine learning, methamphetamine, prediction

## Abstract

**Background:**

Craving is a fundamental aspect of substance use disorder (SUD), traditionally assessed through subjective self‐report measures. To develop more objective assessments, we created a brain‐based marker to predict craving based on machine learning approaches using functional magnetic resonance imaging (fMRI) drug cue reactivity data from 69 participants with methamphetamine use disorders.

**Methods:**

To predict craving intensity (rated on a 1–4 scale), we developed a modeling pipeline in which multiple feature selection methods (ANOVA, PCA) and regression algorithms (linear regression, Lasso, Elastic Net, Random Forest, and XGBoost) were evaluated. Model performance was assessed using subject‐level 5‐fold cross‐validation plus a 20% hold‐out test set. PCA combined with linear regression yielded the best performance in terms of Root Mean Squared Error (RMSE) while maintaining interpretability. Statistical significance was tested via permutation tests. Model weights were back‐projected to voxels and summarized in the Brainnetome atlas. In addition, the model successfully classified high and low craving levels and distinguished cue types (neutral vs. drug) based on fMRI data.

**Results:**

The model achieved an RMSE of 0.983 ± 0.026 (standard deviation) and a mean Pearson correlation of 0.216, with strong generalization evidenced by an out‐of‐sample RMSE of 0.985 and statistical significance (p < 0.026; effect size (Cliff's Delta) = 0.715; statistical power = 0.639). Key neurobiological signatures included the parahippocampal gyrus, superior temporal gyrus, medioventral occipital cortex, and amygdala (positively associated with craving), as well as the inferior temporal gyrus (negatively associated). Classification of high versus low craving levels yielded an AUC‐ROC of 0.684 ± 0.084 (out‐of‐sample AUC‐ROC = 0.714), with significant separation (p < 0.04; Cliff's Delta = 0.831). In addition, classification of cue types (neutral vs. drug) achieved an AUC‐ROC of 0.692 ± 0.090 (out‐of‐sample AUC‐ROC = 0.693), with p < 0.002, Cliff's Delta = 0.896, and statistical power = 0.800, highlighting the robustness of the model.

**Conclusion:**

These findings underscore the potential of neuroimaging and machine learning to provide objective, data‐driven insights into the neural mechanisms underlying subjective experience of craving and to inform future clinical applications in SUD.

## Graphical abstract

Brain responses to drug cues measured with fMRI, combined with craving self‐reports, were used to train a machine learning model that predicted craving intensity and classified high versus low craving states. These findings highlight the promise of neuroimaging‐based biomarkers for advancing personalized interventions in addiction treatment.

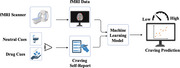

## 1 Introduction

Craving, characterized as an intense desire to consume drugs or food, is a fundamental driver of substance use disorder (SUD), contributing to the compulsive drive to seek drugs despite negative consequences (Koob and Volkow 2010; Tiffany and Wray 2012). It is a potent predictor of relapse (Sinha 2013; Weiss et al. 1995) and has thus become a major focus of addiction research. Functional MRI of drug cue reactivity (FDCR) is a technique in addiction research to understand how the brain responds to drug‐related cues. It involves exposing individuals to drug‐related stimuli (such as images, sounds, or even drug) while capturing brain activity using fMRI. The goal is to identify neural circuits that are activated in response to these cues, as they are associated with craving and relapse in SUDs (Addiction Cue‐Reactivity Initiative (ACRI) Sangchooli et al. 2024; Ekhtiari et al. 2022; Pollard et al. 2023). This method has also been expanded to explore the brain's response to food‐related cues, demonstrating its utility in studying craving mechanisms across substance and non‐substance domains (Field and Cox 2008; Koban et al. 2023).

Research in neuroscience has consistently shown that drug cues elicit activity in several key brain regions. They include the ventromedial prefrontal cortex (vmPFC) and ventral striatum which are known for their roles in reward processing and decision‐making, and the amygdala and hippocampus which are crucial for emotional regulation and memory, especially in response to drug cues (Kilts et al. 2004; Koob and Volkow 2010; Volkow et al. 1997; Zilverstand et al. 2018). The insula has been known for its involvement in interoceptive awareness, helping individuals detect internal bodily states associated with craving (Naqvi and Bechara 2010). These regions collectively form the foundation of the neurocircuitry of addiction, emphasizing both the sensory and emotional components of craving (Koob and Volkow 2010; Naqvi and Bechara 2010; Volkow et al. 2016).

Despite these advancements, our understanding of the neural basis of craving remains incomplete. Current neuroimaging literature highlights the involvement of various brain regions in craving (Volkow et al. 2016; Zilverstand et al. 2018; Seo et al. 2011). However, many studies fail to implement robust predictive modeling techniques, such as cross‐validation, which limits the generalizability of their findings. Instead, they often rely on correlation analyses between subjective craving scores and activity or connectivity in specific brain regions, which are prone to overfitting and circularity in feature selection (Kriegeskorte et al. 2009). These limitations undermine the reliability of population‐level inferences and hinder the development of practical neuroimaging‐based biomarkers. Machine learning approaches offer a solution by enabling the integration of high‐dimensional brain activity patterns into predictive models that can generalize across individuals and contexts. Proper cross‐validation within these models is essential for testing predictive power, increasing replicability, and facilitating the translation of neuroimaging findings into real‐world applications, such as diagnostic tools or personalized treatment interventions. This underscores the urgent need for data‐driven methodologies that leverage machine learning to provide sensitive, specific, and generalizable measures of craving.

Recently, there has been significant progress in leveraging machine learning techniques for craving prediction. Some studies utilized connectome‐based predictive modeling (CPM), a machine learning framework designed to identify functional connectivity patterns predictive of craving in various addictions (Antons et al. 2023; Garrison et al. 2023; Shen et al. 2017). Unlike traditional correlation‐based analyses, CPM employs robust cross‐validation to ensure the generalizability of its findings, making it a more reliable approach for understanding the network‐level mechanisms of craving. Similarly, Koban et al. (2023) employed LASSO‐PCR (Least Absolute Shrinkage and Selection Operator‐Principal Component Regression) to develop the neurobiological craving signature (NCS). This activity‐based approach identifies a reproducible pattern of brain activity involving the vmPFC, ventral striatum, temporal/parietal association areas, cingulate cortex, and mediodorsal thalamus. Their study covered individuals with cocaine use disorder, alcohol use disorder, and smoking addiction, using drug and food‐related visual cues to train and validate their models. The use of study‐stratified 10‐fold cross‐validation ensured robust predictive accuracy, addressing some limitations of traditional methods. While these studies highlight the potential of machine learning to uncover reproducible brain‐based markers of craving, their generalizability across specific substances, such as methamphetamine, and individual variability remains an area for further development. Recent neurocomputational frameworks conceptualize craving as arising from dysregulated prediction‐error and salience attribution processes (Koob and Volkow 2010; Naqvi and Bechara 2010). Network‐based models highlight imbalance between the reward, salience, and control networks. Our voxel‐based approach complements these frameworks by capturing distributed patterns of activation.

Our current study seeks to build upon this body of work by using FDCR from 69 participants with methamphetamine use disorder (MUD) and employing machine learning models to train a predictor being able to predict subjective craving levels based objective neuroimaging data. This will enable us to create detailed maps of brain activity that predicts subjective response to drug cues. These findings could provide opportunities to use fMRI markers as valid measure of craving to monitor response to various interventions and inform targeted interventions for SUD such as transcranial magnetic stimulation (TMS) (R. Chen et al. 1997; Hanlon et al. 2015; Jansen et al. 2015; Pettorruso et al. 2020), transcranial direct current stimulation (tDCS) (Chan et al. 2024; J. Chen et al. 2020) and deep brain stimulation (DBS) (Kuhn et al. 2014; Luigjes et al. 2012).

## 2 Methods

This section outlines the procedures for data collection, imaging protocols, and the analytical framework applied to predict craving intensity from fMRI data. Details on participant selection, imaging settings, data preprocessing, and statistical analyses are provided in the following sections.

### 2.1 Participants

A total of 69 male participants (mean age ± one standard deviation = 35.86 ± 8.47 years), all diagnosed with MUD in their abstinence phase, were recruited for this study. These participants were admitted to the 12&12 Center, a residential abstinence‐based treatment facility in Tulsa, Oklahoma. They were either part of the residential program or its aftercare transitional living programs. They underwent the FDCR task at Laureate Institute for Brain Research (LIBR), in Tulsa, Oklahoma.

The study included participants who met the following conditions: (1) they spoke English, (2) had been diagnosed with MUD within the last year, (3) were enrolled in a residential program designed to support abstinence from methamphetamine, (4) had maintained at least 1 week of abstinence prior to the study, and (5) demonstrated the ability and willingness to engage in the informed consent process.

Individuals were excluded if they (1) were unwilling or unable to complete essential parts of the study, including MRI scanning (e.g., due to claustrophobia), drug cue rating, or behavioral evaluations; (2) reported over 6 months of methamphetamine abstinence; (3) had a diagnosis of schizophrenia or bipolar disorder based on a MINI interview; (4) exhibited active suicidal thoughts, determined either by self‐report or through assessment by study staff at any stage of the study; or (5) had a positive screening for amphetamines, opioids, cannabis, alcohol, phencyclidine, or cocaine, as confirmed by breath or urine testing.

### 2.2 fMRI Task

**FIGURE 1:**
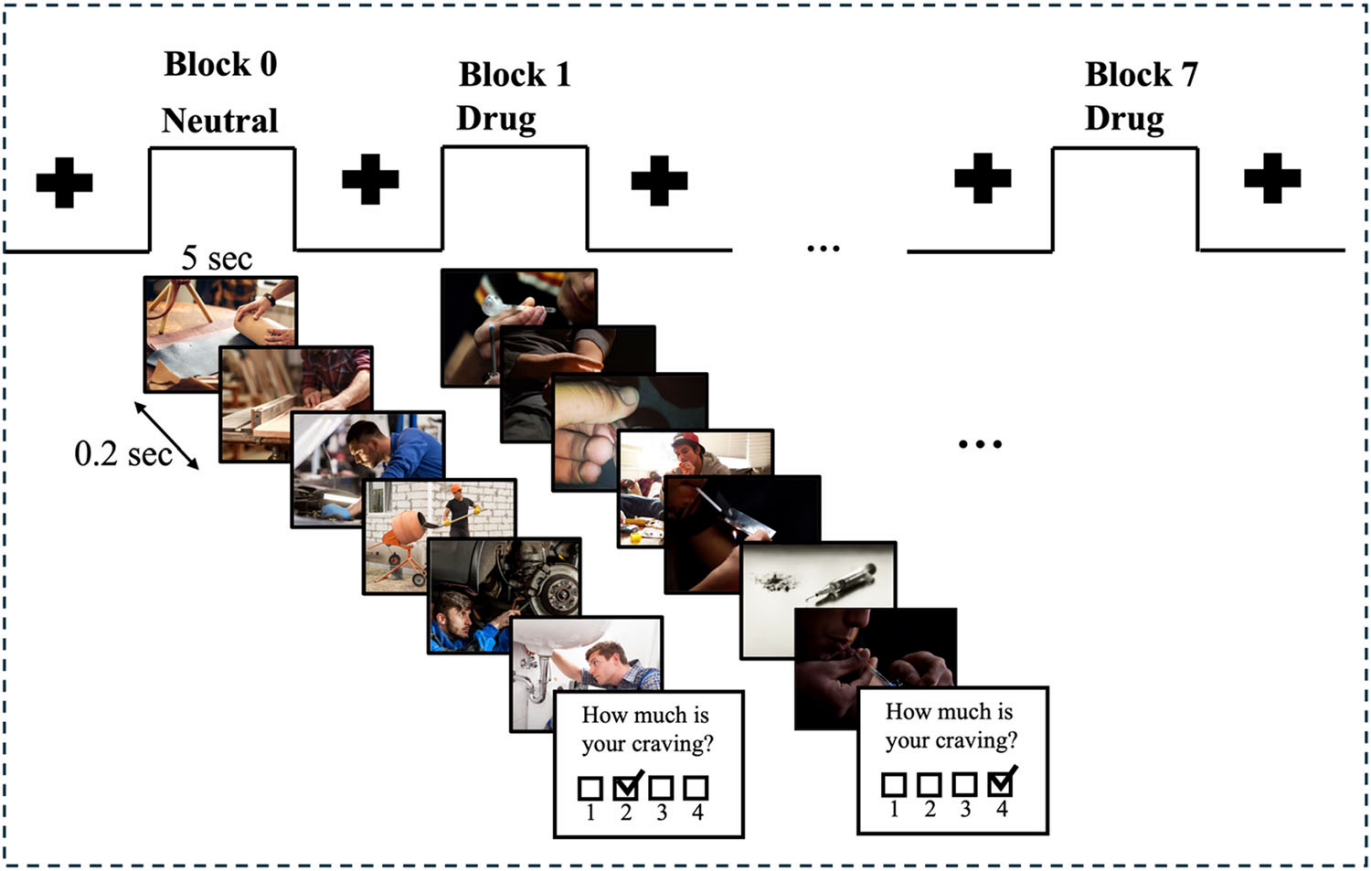
fMRI drug cue reactivity task. The task included alternative blocks of neutral and drug cues (images). Each block consisted of six images each displayed for 5 s with a 0.2 s inter‐stimulus interval resulting in a total block duration of 31 s. A visual fixation point was presented for 8–12 s between consecutive blocks. At the end of each block a self‐report was collected which participants were asked to rate their current meth craving level on a 1–4 rating scale (1: *lowest* to 4: *highest*).

All participants in this study completed the same FDCR task structure as described in (Ekhtiari et al. 2016). During the fMRI task, participants were shown alternating blocks of drug‐related (methamphetamine) and neutral cues. Each block consisted of six images, each displayed for 5 s with a 200 ms blank screen between them, making the total duration of each block 31 s. After each block, participants rated their craving based on the question “How much craving do you have right now?” The craving scale was from 1 to 4, with 1 indicating “*No Urge*” and 4 indicating “*Strong Urge*.” A limitation of this study is the use of a 4‐point self‐report craving scale. While brief Likert‐type ratings are commonly employed in cue‐reactivity paradigms to capture immediate subjective responses (Koban et al. 2023), the restricted granularity of our scale may have reduced sensitivity to individual differences. Moreover, unlike validated multi‐item craving questionnaires, such single‐item ratings do not provide psychometric reliability estimates. Future work would benefit from employing validated, higher‐resolution craving measures to improve construct validity and allow for stronger inferences about the neural correlates of craving. The interval between blocks varied between 8 and 12 s, and the blocks alternated between drug and neutral, starting with a neutral block. A total of eight blocks were presented, with four of each type. The total scan time for this task was approximately 6.5 min (Figure 1).

### 2.3 Image Acquisition

The MR images were collected using two General Electric (GE) MRI 750 3T scanners located at LIBR. The FDCR task lasted 6 min and 32 s, with scan parameters as follows: repetition time/echo time (TR/TE) = 2000/27 ms, field of view (FOV)/slice = 240/2.9 mm, 128× 128 matrix resulting in 1.875 mm × 1.875 mm × 2.9 mm voxels, across 39 axial slices with 196 repetitions. High‐resolution structural images were obtained using an axial T1‐weighted Magnetization Prepared Rapid Gradient Echo (MPRAGE) sequence, with parameters including TR/TE = 5/2.012 ms, FOV/slice = 240 mm × 192/0.9 mm, 256× 256 matrix generating 0.938 mm × 0.928 mm × 0.9 mm voxels, and 186 axial slices.

### 2.4 fMRI Data Preprocessing

First‐level processing was performed using Analysis of Functional NeuroImages (AFNI), which involved several steps: discarding the first three pre‐steady state images, despiking, correcting for slice timing, realigning the images, transforming them to MNI space, and applying 4 mm Gaussian full‐width‐half‐maximum (FWHM) smoothing. Following this, regression analysis was conducted, incorporating nuisance regressors for the first three polynomial terms and the six motion parameters. TRs with the Euclidean norm of the derivative of the six motion parameters being greater than 0.3 points were removed. After preprocessing, a general linear model (GLM) was applied to obtain the beta coefficients. This processed data was subsequently used to train a machine learning algorithm.

### 2.5 Data Characteristics

The beta coefficients derived from the fMRI data presented several analytical challenges. These coefficients, which estimate neural activity associated with specific task conditions, exhibited no readily discernible patterns, making it difficult to identify meaningful clusters or activation profiles (see Figure S1). In addition, the dataset was characterized by a relatively small sample size and extremely high dimensionality (over 1 million voxels), with a substantial proportion of zero values—further complicating the analysis. Craving ratings showed clear differentiation between drug and neutral cues, with overall moderate variability. Detailed summary statistics are presented in Figure S2.

### 2.6 Data Analysis Pipeline

**FIGURE 2:**
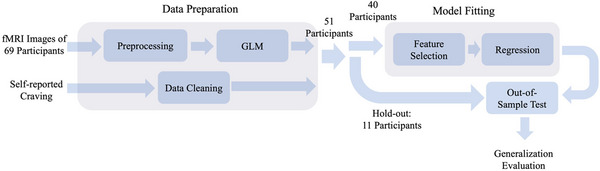
Analysis pipeline. fMRI images and self‐reported craving levels from 69 participants are processed in the data preparation unit. The fMRI data is preprocessed, and a general linear model is applied to generate beta coefficients for data analysis. At the same time, participants with missing or incomplete data (18 participants) are removed. Subsequently, 20% of the data (11 participants) is set aside as an independent hold‐out test set, while the model is fitted using the remaining 80%, comprising approximately 40 participants. The model fitting includes a feature selection step, where the high‐dimensional data is projected to a lower dimension, followed by training a regression model. Finally, the model is tested on the unseen portion of data to evaluate its generalizability.

In our data analysis pipeline (Figure 2), we employed several robust methods to ensure the reliability and validity of our results. We utilized 5‐fold cross‐validation while making sure that the splitting of data to train and test was done in a subject‐level manner. In other words, we made sure the data belonging to the same subject was assigned to the same split. This avoids leaking the subjects’ information to other splits and makes sure a reliable model development (Kohavi 1995). In addition, we used hold‐out testing by setting aside 20% of the data as a hold‐out test set to evaluate the model's performance on unseen data.

To assess the model performance across varying degrees of craving intensity, we stratified metrics based on different craving levels. In other words, the performance was evaluated separately for each craving level and then the results were averaged to obtain an overall performance score. This way, we don't have to worry about the craving levels being imbalanced in each split as we already used the stratified metrics. We initially explored a mixed‐effects model to account for within‐subject variability across multiple trials per participant. However, the objective of machine learning—where models are trained to generalize to unseen subjects—fundamentally differs from the intent of mixed‐effects modeling, which explicitly captures subject‐specific variations. The limited per‐subject sample size and minimal variance explained by random effects further reduced its effectiveness. In contrast, standard machine learning approaches, such as linear regression, optimize fixed effects across the entire dataset, enhancing generalizability and predictive performance for new subjects.

We also conducted permutation test to evaluate the significance of our results, involving the repeated shuffling of craving levels (1000 times) to generate a distribution of outcomes under the null hypothesis, which allowed us to determine the statistical significance of our findings. These approaches were crucial in developing a reliable and generalizable predictive model for craving intensity. It is important to note that we did not apply a voxel‐wise multiple comparisons correction to the regional back‐projection results. While PCA and permutation testing mitigate some of these concerns, the identification of specific regions should be considered exploratory and requires replication in larger samples.

### 2.7 The Model and Hyperparameter Tuning

We have implemented a comprehensive two‐step process for feature selection, followed by model training (Figure 2). Initially, we evaluated PCA and analysis of variance (ANOVA) for feature selection. The features correspond to distinct voxel locations within the brain. Subsequently, we assessed five regression algorithms, including Multivariate Linear Regression (Anderson 1958), Ridge Regression (Hoerl and Kennard 1970), Lasso Regression (Tibshirani 1996), Elastic Net (Zou and Hastie 2005), Random Forest (Breiman 2001), and XGBoost (T. Chen and Guestrin 2016), to identify the optimal model that minimizes RMSE. These methods were selected based on their complementary strengths in handling high‐dimensional data and regularization. Multivariate Linear Regression serves as a baseline model to evaluate the effectiveness of other approaches. Ridge and Lasso Regression are well‐suited for reducing overfitting in high‐dimensional data through L2 and L1 regularization, respectively. Elastic Net combines these two penalties to handle correlated features effectively. Random Forest, a robust ensemble learning method, was chosen for its ability to model non‐linear relationships and inherent feature importance evaluation. Finally, XGBoost, a state‐of‐the‐art gradient boosting algorithm, was included for its efficiency and strong performance in a wide range of regression tasks. Moreover, before feature selection, we eliminate features with very low variance (Var < 0.001). In addition, we ensure that the data is centered using a standard scaler before applying PCA. To determine the optimal hyperparameters, we employ the GridSearchCV (Pedregosa et al. 2011) class from the scikit‐learn Python library, calculating RMSE for different scenarios (see Table S1).

In the optimized pipeline, the data used in model fitting was projected into a lower‐dimensional space by multiplying it with the eigenvector matrix (V) derived from PCA. This projected data was then combined with the linear regression coefficient vector (B) to predict craving levels. The resulting product VB represents a key neurobiological signature of craving (Koban et al. 2023) forming a one‐dimensional vector that indicates the contribution of each voxel to the craving process. To identify the activated subregions more closely, Brainnetome atlas parcellation was applied to extract 246 subregions, each containing a cluster of voxels (Fan et al. 2016). Each voxel was assigned to its corresponding Brainnetome subregion, and the average VB value for all voxels within each subregion was calculated.

In general, the craving intensity, particularly whether it is high or low is of primary interest. To enhance model interpretability, we classified craving levels into high and low and focused on the most extreme craving states. Here, the steps of the algorithm align with those implemented in the regression algorithm, with an additional mapping step. Here, the proper number of PCA components was determined to be 50 due to lower number of samples. During the mapping phase, the regression algorithm's outcomes are categorized into high or low levels, reflecting the predicted outcomes. Given that the predicted results of the regression are not uniformly distributed between values of 1 and 4, and that this distribution varies in each iteration of cross validation, we need to optimize the threshold level for the classification. We utilized *F*_1_ score as the metric for threshold selection due to the label imbalance observed in each iteration, ultimately selecting the threshold that maximizes the *F*_1_ score at each iteration. These thresholds were optimized for methodological performance (*F*_1_ score) and should not be interpreted as clinically validated cut‐offs.

To evaluate the robustness of our algorithm, we trained a predictor to classify cue types as neutral or drug‐related (defined as 0 and 1, respectively). We again employed the same pipeline as before with the addition of a step mapping the regression output to 0 and 1 with the threshold equal to 0.5 and the number of components of PCA equal to 100.

## 3 Results

We have considered three primary aims for this project. First, aimed to predict the intensity of craving. Following the pipeline in Figure 2, we optimized the model and proved that the result is statistically significant. This identified a brain map highlighting the contribution portion of each subregion. Next, we developed a classification algorithm to be able to distinguish between the high and low craving based on fMRI images and proved its statistical significance. Lastly, we developed an algorithm differentiating the cue types and again confirmed its significance.

### 3.1 Craving Intensity Prediction

Our analysis shows that PCA followed by linear models, including linear regression, Lasso, and Elastic Net, with slightly similar performance, outperform non‐linear models in both feature selection (ANOVA) and regression algorithms (Random Forest and XGBoost). Among these, linear regression was selected as the final model due to its optimal balance of prediction accuracy and interpretability. Since dimensionality was already reduced via PCA, additional regularization from Lasso or Elastic Net provided minimal benefit. The simplicity and transparency of linear regression also facilitated back‐projection of weights onto voxel space, enabling clearer identification of neurobiological contributors to craving. In conclusion, PCA with 100 components and linear regression emerged as the best approach for this analysis (Table S1).

The results presented in Table 1 demonstrate the statistical comparison between the Proposed Algorithm and the Null Hypothesis across three key performance metrics: RMSE, MAE, and correlation coefficient. The Shapiro–Wilk test was used to assess normality, revealing that the correlation coefficient followed a normal distribution, while RMSE and MAE did not. Given this, Cohen's *d* was used as the effect size measure for correlation, and Student's *t*‐test was employed to compute the confidence interval (95% CI = [0.209, 0.222]). The corresponding *p* value (0.050) and effect size (1.826) indicate a strong difference between the two methods, with a statistical power of 50.5%.

**TABLE 1:** Statistical comparison of the proposed algorithm and the null hypothesis for the prediction of craving level. The mean, standard deviation (Std Dev), 95% confidence interval (CI), *p* value, effect size, and statistical power are reported. The out‐of‐sample performance is also included to evaluate generalization. Lower RMSE and MAE values indicate better predictive performance, while higher Pearson correlation show better performance.

Metric	Mean	Std Dev	95% CI	*p*‐value	Effect size	Statistical power (%)	Out‐of‐sample
RMSE	0.983	0.026	(0.98, 0.984)	0.028	0.715	63.9	0.985
MAE	0.94	0.014	(0.939, 0.941)	0.008	0.836	74.6	0.976
Correlation	0.216	0.112	(0.209, 0.222)	0.05	1.826	50.5	0.108

Conversely, RMSE and MAE exhibited non‐normal distributions, necessitating the use of Cliff's Delta for effect size and bootstrap resampling for confidence interval estimation. For RMSE, the 95% CI (0.980, 0.984) was obtained via bootstrapping, with an effect size of 0.715, a *p* value of 0.028, and a statistical power of 63.9%. Similarly, for MAE, the 95% CI (0.939, 0.941) was computed through bootstrapping, with an effect size of 0.836, a *p* value of 0.008, and a statistical power of 74.6%.

In addition, statistical significance (*p* value) and power were determined by counting from the 5% critical value threshold to evaluate the reliability of the observed differences. The out‐of‐sample performance further supports these findings, with RMSE and MAE showing robust generalization (0.985 and 0.976, respectively), whereas correlation exhibited a lower out‐of‐sample predictive value (0.108).

**FIGURE 3:**
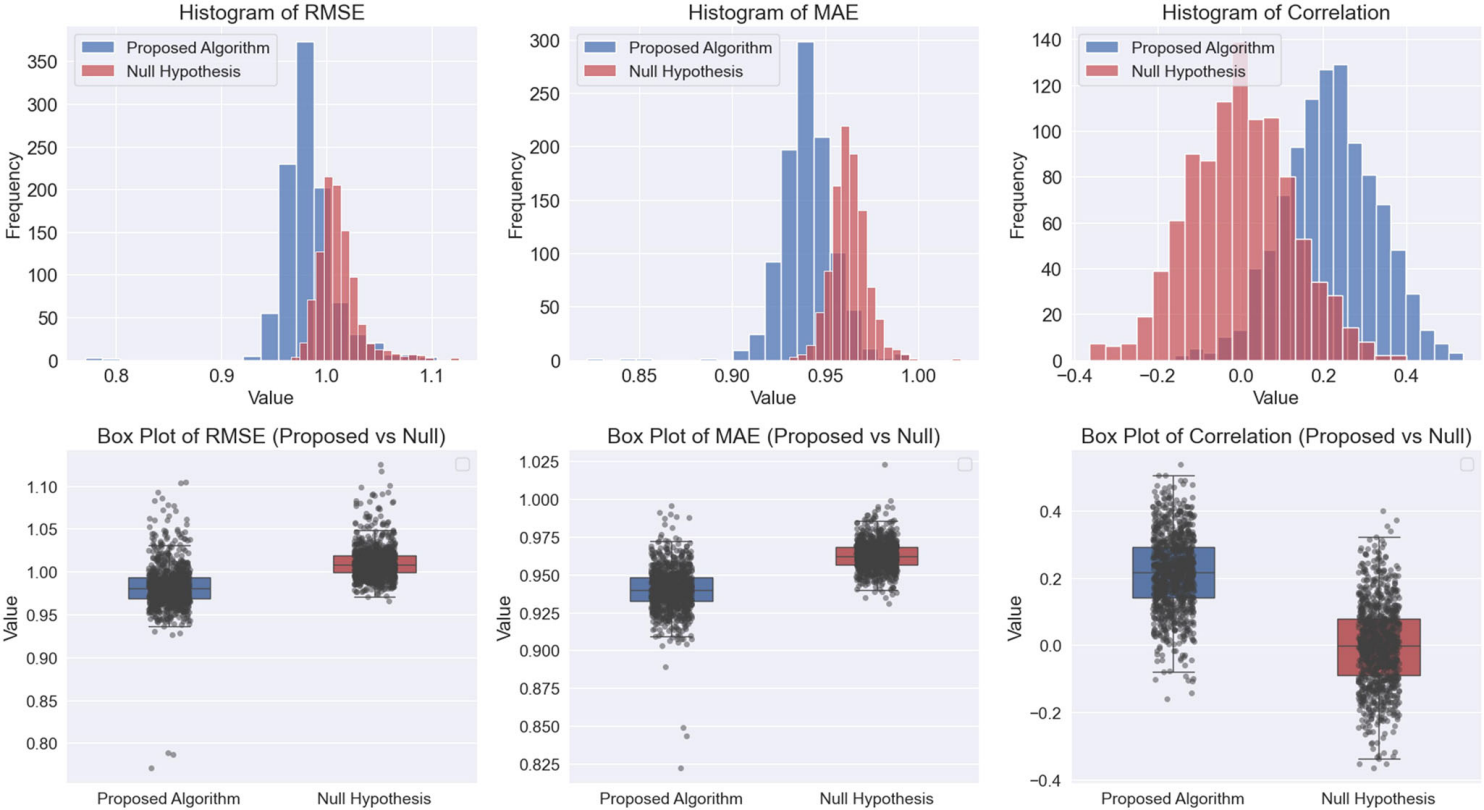
Comparison of the results between the proposed algorithm and the null hypothesis across three evaluation metrics: RMSE, MAE, and correlation coefficient. Top Row: The histograms illustrate the distributions of results for the proposed algorithm (blue) and the null hypothesis (red). Bottom Row: The box plots provide a comparative visualization of distribution spread and variability. Each box represents the interquartile range (IQR) (middle 50% of data), with the central line indicating the median. The whiskers extend up to 1.5 times the IQR, and data points beyond these whiskers are considered outliers, plotted as individual dots.

Figure S3 outlines the out‐of‐sample results for the regression problem. For each participant, only the minimum and the maximum self‐reported craving levels were considered. In cases with multiple instances of minimum or maximum cravings, only the first occurrence was used. The fitted normal distribution of the outcomes and associated box plots are presented in Figure 3.

**FIGURE 4:**
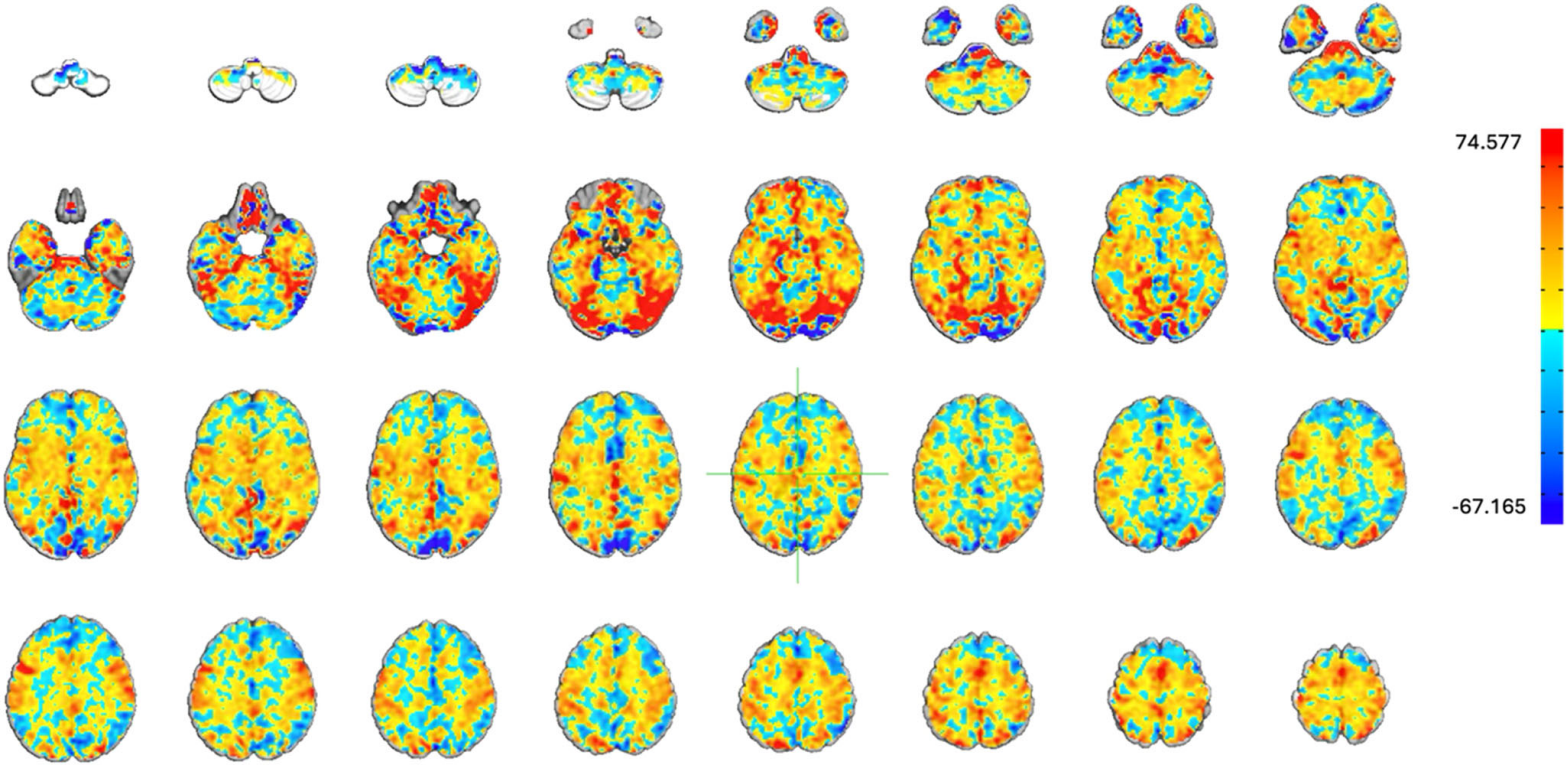
Neurobiological signature of craving. This map illustrates the contribution of each region to the prediction of subjective craving intensity. The red regions represent areas with positive contributions, while the blue regions indicate areas with negative contributions.

**FIGURE 5:**
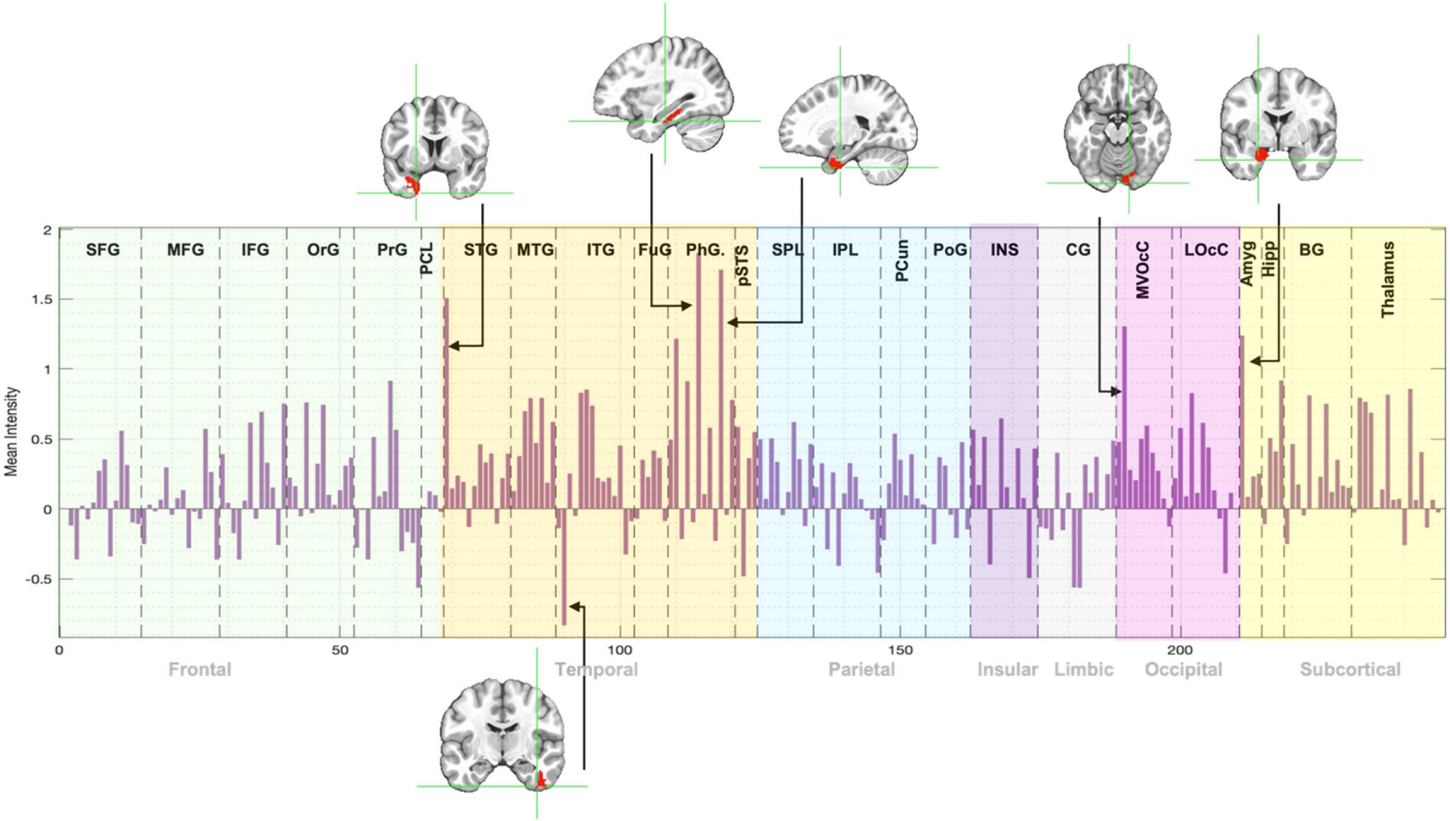
Brainnetome subregions bar plot. An illustration of the involvement of various brain subregions from the Brainnetome atlas in relation to craving. Each bar represents a specific subregion, with the height indicating the strength of contribution with craving. Key neurobiological signatures identified were the parahippocampal gyrus, superior temporal gyrus, medioventral occipital cortex, and amygdala, which showed positive contributions to craving, and the inferior temporal gyrus, which exhibited a negative contribution to craving.

The neurobiological signature of craving associated to our algorithm is illustrated in Figure 4. Regions with higher values amplify the corresponding beta coefficients, while regions with lower values attenuate them. For visualization purposes, the signature was normalized to align with the norm of the average beta coefficients derived from the fMRI data. Figure 5 displays the Brainnetome bar plot, showing that the parahippocampal gyrus, superior temporal gyrus, medioventral occipital cortex, and amygdala contributed most to craving, while the inferior temporal gyrus (ITG) exhibited the most negative contribution.

### 3.2 High and Low Craving Classification

The performance of the proposed algorithm compared to the null hypothesis is presented in Table 2. RMSE, accuracy, and area under the receiver operating characteristic curve (AUC‐ROC) were evaluated to assess prediction and classification performance.

**TABLE 2:** Statistical comparison of the proposed algorithm and the null hypothesis for the classification of high and low craving across RMSE, accuracy, and AUC‐ROC metrics. The mean, standard deviation, 95% confidence interval (CI), *p* value, Cliff's Delta effect size, and statistical power are reported. The out‐of‐sample performance is also included to evaluate generalization. Lower RMSE values indicate better predictive performance, while higher accuracy and AUC‐ROC values reflect improved classification capability.

Metric	Mean	Std Dev	95% CI	*p*‐value	Effect size (Cliff's Delta)	Statistical power (%)	Out‐of‐sample
RMSE	1.459	0.024	(1.457, 1.460)	0.044	0.821	41.3	1.463
Accuracy	0.674	0.108	(0.668, 0.681)	0.07	0.753	30.3	0.703
AUC‐ROC	0.684	0.084	(0.679, 0.689)	0.04	0.831	43.3	0.714

For classification accuracy, the proposed algorithm demonstrates some improvements in the evaluated metrics compared to the null hypothesis. It is important to note that all metric distributions follow a non‐normal distribution; therefore, we employ non‐parametric or appropriate statistical methods for estimating parameters. The accuracy of 0.674 ± 0.108 indicates that the algorithm's performance exceeds chance level with a moderate margin. The CI of (0.668, 0.681), calculated using bootstrap sampling, supports the reliability of the results though the *p* value of 0.07 suggests moderate statistical significance. The effect size, measured by Cliff's Delta (0.753), indicates a substantial difference; however, the limited statistical power (30.3%) underscores the need for further validation with larger sample sizes. The out‐of‐sample accuracy was 0.703, surpassing the in‐sample accuracy, which suggests strong generalization to unseen data.

The AUC‐ROC, which evaluates the classifier's ability to distinguish between low and high craving states, was 0.684 ± 0.084 for the proposed algorithm. The CI of (0.679, 0.689) confirms the robustness of this metric. The *p* value of 0.04 indicates statistical significance, and the effect size (Cliff's Delta: 0.831) suggests a strong improvement. The statistical power was 43.3%, indicating a limited probability of detecting a true effect with the current sample size. Nonetheless, the out‐of‐sample AUC of 0.714 provides additional support for the model's ability to generalize to unseen data.

**FIGURE 6:**
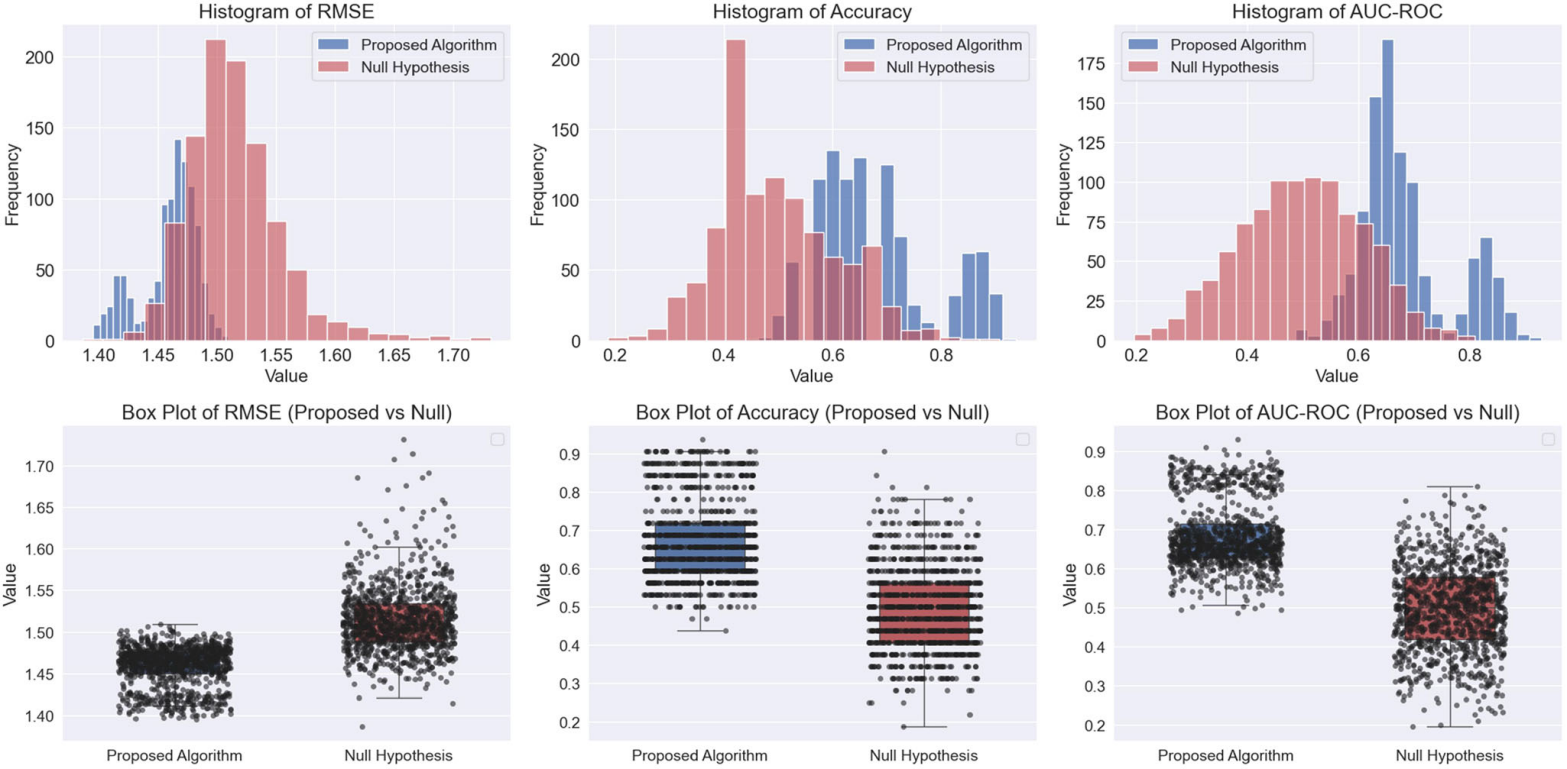
**Illustration of the results of classification algorithm for high and low craving levels**. Comparison of the proposed algorithm and the null hypothesis (permutation test) for RMSE, accuracy, and AUC‐ROC. The top row displays histograms showing the distribution of each metric for both models, with the proposed algorithm in blue and the null hypothesis in red. The bottom row presents box plots illustrating the variability and spread of each metric.

Figure 6 provides a visual summary of these findings, showing clear separation between the proposed algorithm and the null model across RMSE, accuracy, and AUC‐ROC. Overall, the proposed algorithm consistently outperformed the null hypothesis in prediction and classification tasks. The significant improvements in RMSE, accuracy, and AUC‐ROC, along with moderate‐to‐large effect sizes, indicate meaningful differences between the proposed model and the permutation‐based null hypothesis. The higher out‐of‐sample accuracy and AUC further validate the model's generalization ability in craving classification and prediction.

**FIGURE 7:**
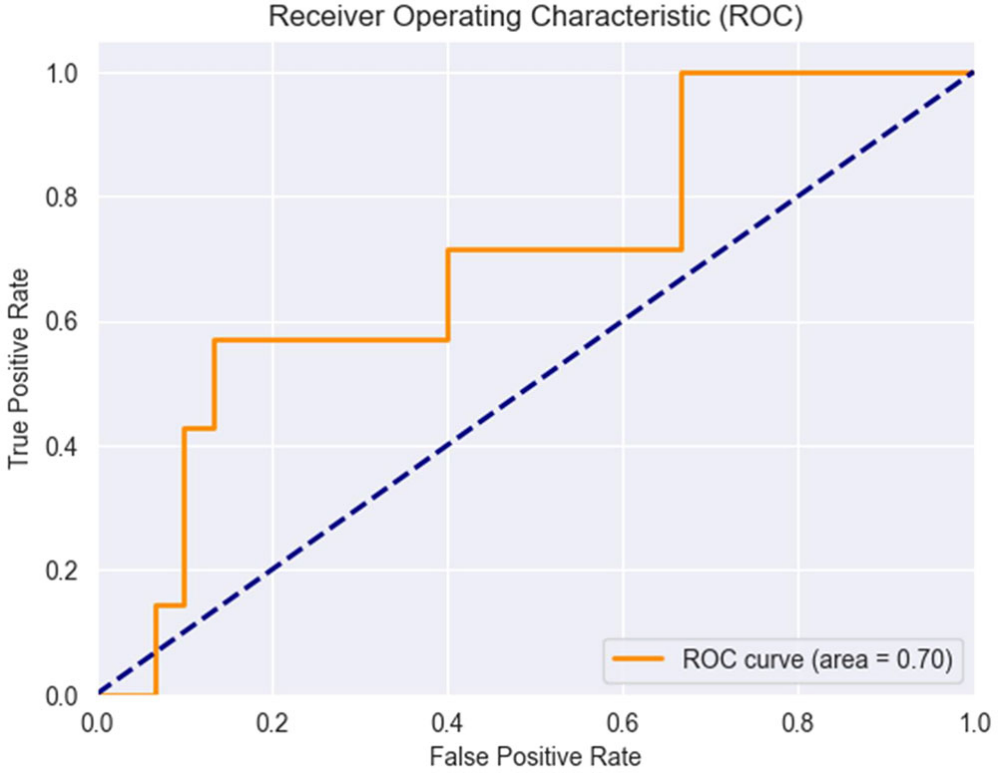
ROC curve for the high and low classification of craving on unseen data. The curve illustrates the trade‐off between the True Positive Rate (TPR) and False Positive Rate (FPR) across classification thresholds. The dashed diagonal line represents the baseline performance of a random classifier. The AUC value of 0.70 reflects moderate discriminative ability. The staircase‐like shape of the curve is due to the small number of participants in the test set, affecting the curve's granularity.

The ROC curve (Figure 7) shows the model's performance on unseen data, with an AUC of 0.70, suggesting moderate discriminatory ability between classes.

### 3.3 Cue Type Classification

Table 3 provides a detailed statistical summary of classifier performance. Since the metrics exhibited a non‐normal distribution, we used Cliff's Delta to estimate effect size and applied bootstrap resampling to compute confidence intervals. The classifier achieves an accuracy of 0.617 ± 0.057, with a Cliff's Delta of 0.863, indicating a substantial effect size. The highest performance is observed in the precision metric (0.723 ± 0.095, Cliff's Delta = 0.900), suggesting that when the classifier identifies a cue type, it is often correct. The AUC‐ROC score of 0.692 ± 0.090 and AUC‐PR of 0.698 ± 0.082 further confirm the model's strong ability to discriminate between cue types. Notably, the *p* values across all metrics are below 0.05, indicating that the observed differences are unlikely due to chance. The statistical power for these tests ranges from 60.5% to 80.0%, reducing the likelihood of Type II errors.

**TABLE 3:** Cue type classification results. Performance metrics of the cue type classifier, including Accuracy, Precision, AUC‐ROC, and AUC‐PR. The results demonstrate the classifier's strong discriminative ability, with significantly higher performance compared to the null hypothesis. The low *p* value, high power, and large effect size further support the statistical significance and generalizability of the model.

Metric	Mean	Std Dev	95% CI	*p*‐value	Effect size (Cliff's Delta)	Statistical power (%)	Out‐of‐sample
Accuracy	0.617	0.057	(0.613, 0.620)	0.02	0.863	60.5	0.614
Precision	0.723	0.095	(0.717, 0.729)	0.02	0.9	68.1	0.727
AUC‐ROC	0.692	0.09	(0.686, 0.697)	0.002	0.896	80	0.693
AUC‐PR	0.698	0.082	(0.693, 0.703)	0.015	0.888	64.8	0.681

**FIGURE 8:**
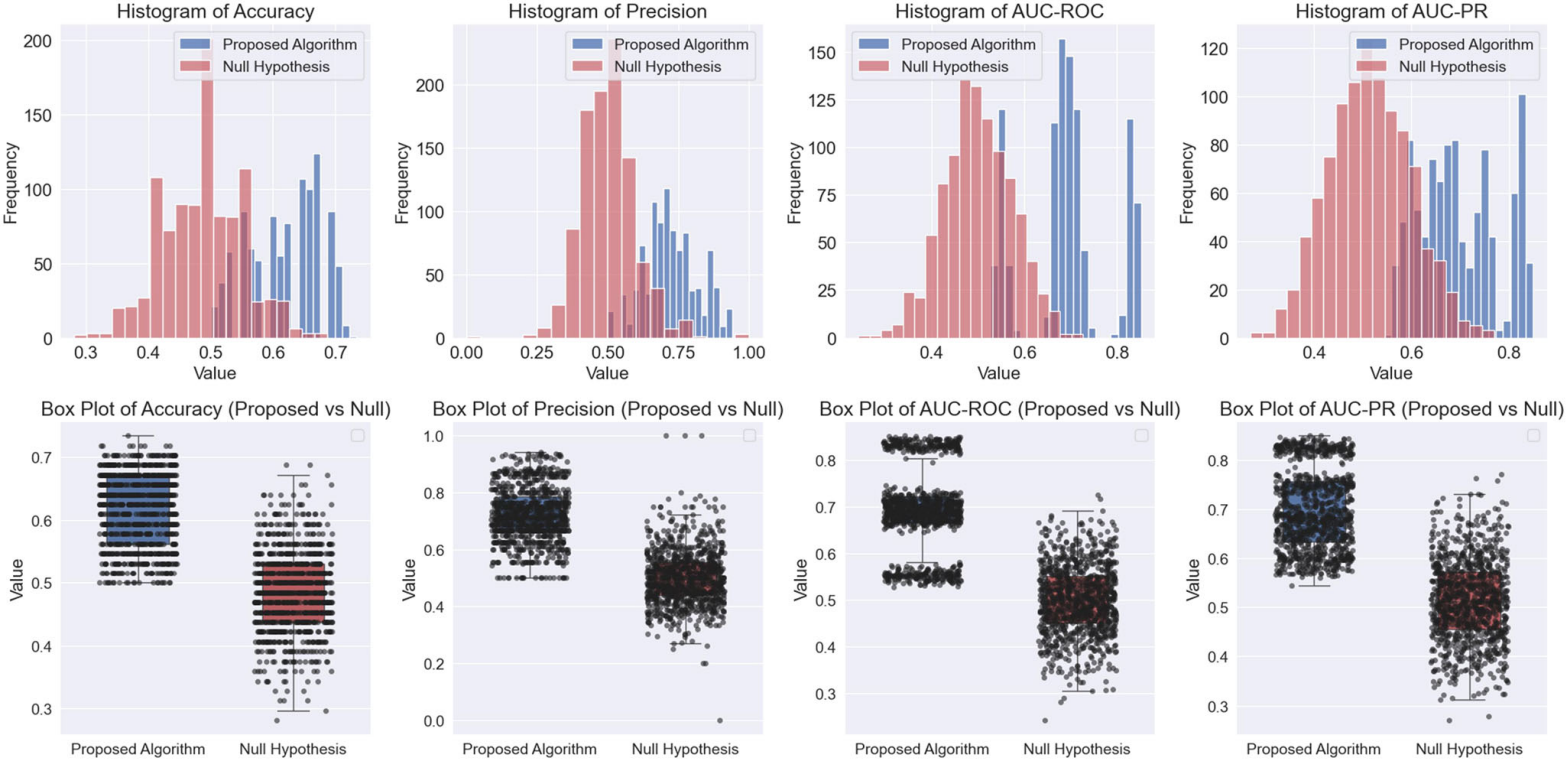
Comparison between the proposed algorithm and the null hypothesis for cue type classification performance. The top row shows histograms of accuracy, precision, AUC‐ROC, and AUC‐PR, highlighting the distribution of each metric. The bottom row presents corresponding box plots, illustrating differences in median values and variability.

**FIGURE 9:**
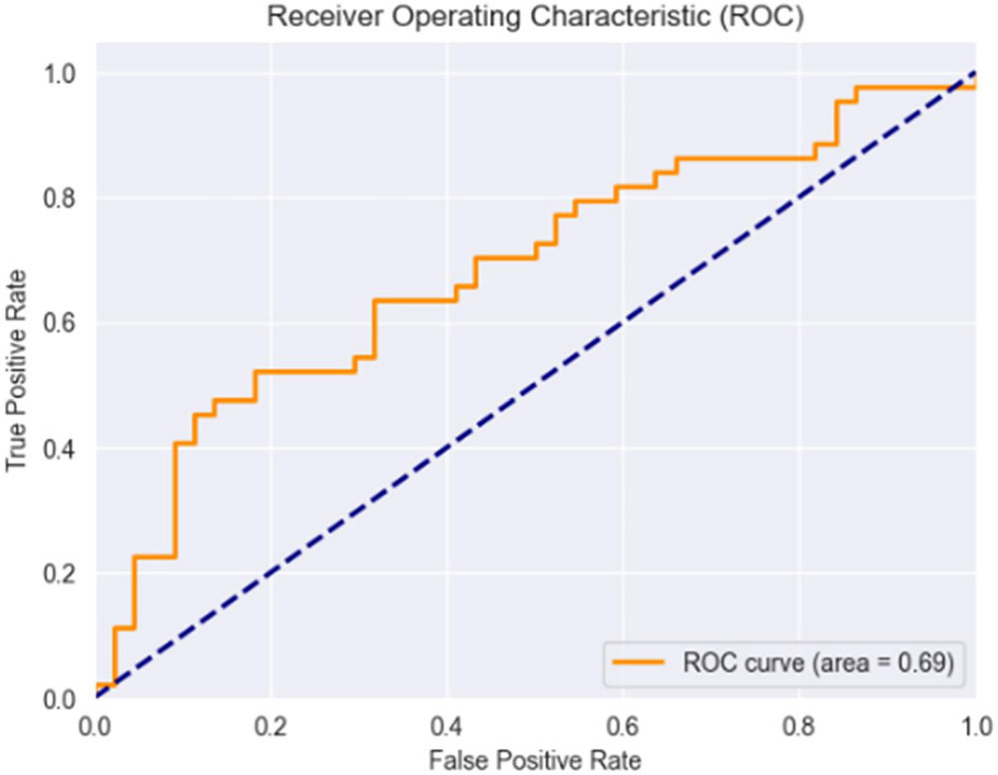
The receiver operating curve of the unseen data (out‐of‐sample ROC) for classifying the cue type. The curve shows the trade‐off between the TPR and FPR at various threshold settings. The dashed diagonal line represents the baseline.

The discriminative power and statistical significance of the classifier, assessed using a permutation test against the null hypothesis, are presented in Figure 8 for various performance metrics. The histograms and box plots illustrate the differences between the proposed algorithm and the null hypothesis across accuracy, precision, AUC‐ROC, and area under the precision‐recall curve (AUC‐PR). The proposed algorithm consistently outperforms the null hypothesis, as seen in the separation of distributions and higher median values across all metrics. Figure 9 illustrates the ROC curve for the classifier on unseen data, demonstrating a moderate ability to distinguish between cue types, with an AUC‐ROC of 0.69.

## 4 Discussion

The results of this study demonstrate that our machine learning pipeline, which includes PCA and linear regression, can reliably and validly predict subjective craving intensity based on objective drug cue reactivity fMRI data. These results enhance our understanding of the neurobiological contributions to craving and underscore their clinical relevance for addiction and related disorders.

It is important to acknowledge that craving is a multidimensional construct, encompassing tonic versus phasic and cognitive versus affective components. Our block‐level cue‐reactivity paradigm most directly assesses phasic affective craving; future studies with ecological momentary assessment and task designs can further dissociate these dimensions.

The comparable metrics for both in‐sample and out‐of‐sample data underscore the generalizability of our model. While the effect sizes were in the moderate to large range, the statistical power was not consistently high, suggesting that additional data may be necessary for more robust validation. It is also important to note that the modest correlation observed in our study (*r* ≈ 0.22, < 5% variance explained) is consistent with prior predictive modeling studies of craving. For example, Garrison et al. (2023) reported predictive correlations ranging from *r* ≈ 0.20–0.41 across different analytic adjustments and samples. These findings suggest that predictive correlations in the 0.2–0.4 range are typical in this emerging area of research, reflecting both the subjective variability of craving and the modest signal‐to‐noise ratio of fMRI. Our results therefore contribute to a growing body of work demonstrating feasibility, while underscoring the need for larger samples and refined methods to achieve clinical utility. The positive trend in the slope of the lines connecting minimum and maximum craving points suggests that the model captures the variations in craving intensity effectively (Figure S3). This reliable differentiation between extremes in craving levels supports the potential application of the model in understanding and predicting craving dynamics in SUDs.

By identifying the brain activity map, specifically the roles of the parahippocampal gyrus, superior temporal gyrus, medioventral occipital cortex, amygdala (positive contribution), and ITG (negative contribution), we provide valuable insights into the brain regions that influence craving intensity. Note that these regional findings should be considered exploratory because the regression coefficients derived from PCA‐reduced data are not directly interpretable as mechanistic activation maps. The parahippocampal gyrus and amygdala play a well‐established role in craving, as these regions are crucial for emotional processing, memory, and responses to cues—all central mechanisms in craving (Breiter et al. 1997; Koob and Volkow 2010). Findings in the medioventral occipital cortex and ITG may partly reflect visual cue processing rather than craving‐specific processes, and should be interpreted cautiously. Their involvement aligns with findings in the literature, particularly in addiction studies. Superior temporal gyrus is involved in sensory integration and emotional regulation (Bigler et al. 2007; Radua et al. 2010), which can also contribute to craving responses. Medioventral occipital cortex is linked to visual processing, potentially related to visual cues triggering craving (Kanwisher and Yovel 2006; Sabatinelli et al. 2009). The negative contribution of ITG in craving could reflect inhibitory or regulatory processes, but this might require further investigation to align with existing studies. The ITG is primarily associated with visual object recognition and processing within the ventral visual stream (Haxby et al. 1996). Its involvement in craving may be linked to the processing of visual cues related to addictive substances. However, the specific role of the ITG in inhibitory control over craving is not well‐established and needs further investigation.

The algorithm developed in Koban et al. (2023) identified key brain regions associated with craving across multiple groups, including individuals with alcohol use disorder, cocaine use disorder, and cigarette. These regions included the ventromedial prefrontal and cingulate cortices, ventral striatum, temporal/parietal association areas, mediodorsal thalamus and cerebellum. These findings align with our results as both highlight temporal regions suggesting a shared emphasis on regions involved in memory and sensory processing. These functions are crucial for cue‐induced cravings, as memory and sensory cues can trigger cravings. On the other hand, while they emphasize higher‐order areas like the vmPFC, cingulate cortices, and ventral striatum, which are involved in decision‐making, emotional regulation, and reward processing, our results highlight the amygdala, parahippocampal gyrus, and occipital cortex, indicating a stronger focus on emotional response (amygdala) and visual processing (medioventral occipital cortex) related to visual cue‐induced craving.

The developed classifiers for discriminating between high and low craving levels and also distinguishing between neutral and drug‐related cues based on fMRI data highlights their potential utility in clinical settings. These capabilities could inform the development of targeted interventions and personalized treatment plans for individuals with SUDs. While these classifiers performed significantly above chance, their discriminative ability was only moderate (AUC values ∼0.71). Such performance falls below thresholds typically required for clinical utility. We therefore view these findings as early biomarker candidates that highlight feasibility, but which require refinement and external validation in larger samples before clinical application.

To enhance our findings, increasing access to larger datasets could significantly improve the generalizability of results, allowing for more nuanced conclusions. Although safeguards such as subject‐level cross‐validation, a 20% independent hold‐out test set, and permutation testing mitigate overfitting, the modest sample size remains a limitation. Larger multi‐site datasets are essential to establish replicability. A notable limitation is the exclusion of 18 participants due to missing or incomplete data, which reduced the final analytic sample to 51. This smaller sample size further constrained statistical power and increased the risk of overfitting. While this exclusion ensured data quality, it may have introduced selection bias. Future studies with larger and more complete datasets, ideally across multiple sites, will be necessary to confirm the robustness and generalizability of our findings. Incorporating additional covariates, including sex, weight, age, education level, handedness, and socioeconomic status, is essential to reduce potential confounding variables, thereby improving model fit and validity. Furthermore, employing a self‐regulation strategy during data analysis could provide insights into how individual differences influence craving responses and modulation, ultimately yielding a more comprehensive understanding of the underlying mechanisms (Muraven and Baumeister 2000).

Moreover, expanding our investigation to consider the effects of various other substances will allow for a comparative analysis that may uncover unique neurobiological signatures associated with different drugs. Lastly, exploring the impact of food and other sensory cues on craving could illuminate how environmental factors interact with individual neurobiology to shape craving intensity (Field and Cox 2008). Collectively, these approaches not only promise to enhance our findings but also contribute to a more holistic understanding of craving in the context of addiction research.

## 5 Conclusion

We developed a machine learning pipeline that is able to predict the subjective craving based on objective fMRI image. The process involved multiple key steps: first, optimizing the model based on brain functional activity data, followed by identifying the most critical brain activity map. We then categorized the craving levels and cue types based on this pipeline. A vital part of the analysis was ensuring the generalization and statistical significance of the results across participants.

In terms of the identified activity map, several regions showed the most significant impact on craving intensity. The parahippocampal gyrus, superior temporal gyrus, medioventral occipital cortex, and amygdala emerged as having the most positive contribution to craving. Conversely, the ITG showed a negative contribution. This comprehensive pipeline allows for the classification of brain activity patterns related to craving, advancing our understanding of the underlying neurobiological mechanisms. Overall, our study represents a significant step towards objective, neurobiologically‐informed assessments of craving, with implications for improving addiction treatment and outcomes. For further validation, the model should be evaluated in varied populations and settings.

## Author Contributions

H.M.‐D. and H.E. conceived and designed the study. H.M.‐D. performed data analysis and drafted the manuscript. G.S. assisted with data preprocessing and interpretation. K.O.L. provided institutional and academic support. H.E. supervised the project and contributed to data interpretation and manuscript revision. All authors reviewed and approved the final version of the manuscript.

## Ethics Statement

This study was approved by the Western Institutional Review Board (WIRB; Protocol #20171742) and conducted in accordance with the Declaration of Helsinki and all relevant ethical guidelines and regulations.

## Conflicts of Interest

The authors declare no conflicts of interest.

## Peer Review

The peer review history for this article is available at https://publons.com/publon/10.1002/brb3.70991.

## Supporting information




**Supplementary Figure**: brb370991‐sup‐0001‐figuresS1‐S3.docx

**Supplementary Table**: brb370991‐sup‐0001‐tableS1.csv

## Data Availability

The data supporting the findings of this study are available from the corresponding author upon reasonable request.
