# Supplementary material for: Craving Prediction From fMRI Drug Cue Reactivity in Methamphetamine Use Disorder: A Parsimonious Neurobiological Model
Source: Brain Behav. 2025 Oct 20;15(10):e70991. doi: 10.1002/brb3.70991 (PMC12537833; doi:10.1002/brb3.70991)
Supplement: Supplementary file 1 — Supplementary Figure: brb370991‐sup‐0001‐figuresS1‐S3.docx [file BRB3-15-e70991-s002.docx]

**Supplementary Material**

**Title:** Craving Prediction from fMRI Drug Cue Reactivity in Methamphetamine Use Disorder: A Parsimonious Neurobiological Model

Hajar Mahdavi-Doost ^1, 2^, Ghazaleh Soleimani^2^, Kelvin O Lim^2^, Hamed Ekhtiari^2, 3^

^1^Department of Psychology, University of Minnesota, Minneapolis, MN

^2^Department of Psychiatry and Behavioral Sciences, University of Minnesota, Minneapolis, MN

^3^Laureate Institute for Brain Research, Tulsa, OK

**Corresponding Author:**
Hajar Mahdavi-Doost
Email: **hmahdavi@umn.edu**

**Ethics Statement:**
This study was approved by the Western Institutional Review Board (WIRB; Protocol #20171742) and conducted in accordance with the Declaration of Helsinki and all relevant ethical guidelines and regulations.


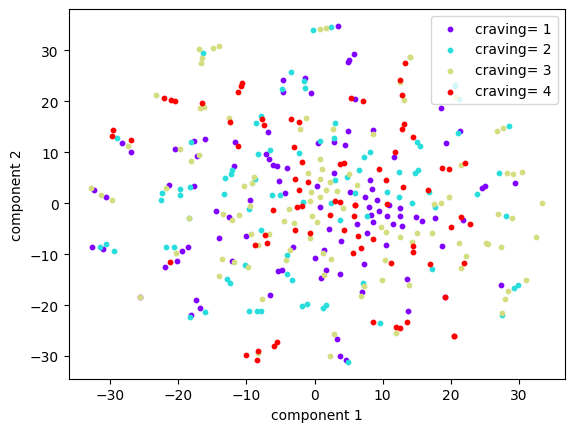


**Supplementary Figure 1. Visualization of the First Two Components of t-SNE (t-Distributed Stochastic Neighbor Embedding) applied to fMRI data.** This 2D representation shows the reduced-dimensional structure of fMRI beta coefficients derived from cue-reactivity tasks. Each point represents a sample, with different colors indicating distinct craving levels. The t-SNE algorithm preserves local relationships, allowing for a qualitative assessment of how neural responses relate to craving intensity.

***
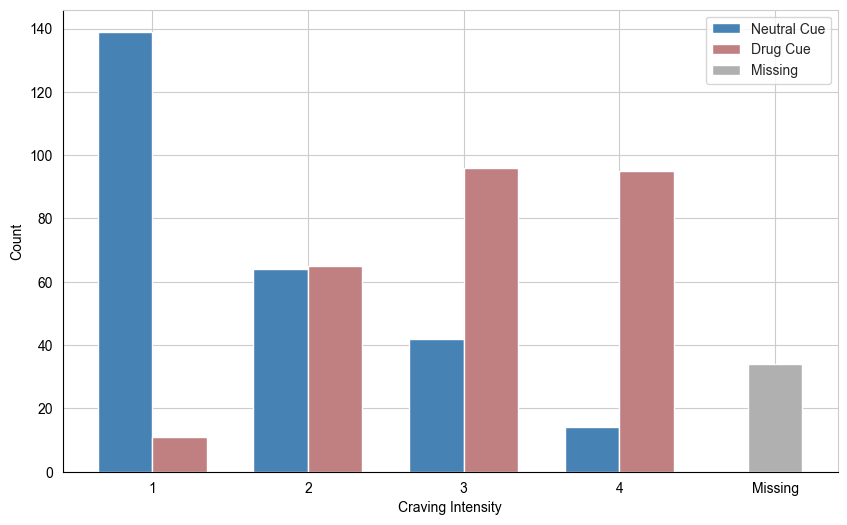
* Supplementary Figure 2. Distribution of self-reported craving intensity for neutral and drug cues, rated on a scale from 1 to 4.** The overall mean craving intensity is 2.37 ± 1.10 (Std Dev), reflecting moderate variability in responses. The median value of 2.0 indicates that half of the responses fall below this level, emphasizing a tendency toward lower craving levels. Neutral cues elicited lower craving ratings (1.76 ± 0.91), while drug cues resulted in higher reported cravings (3.02 ± 0.86), demonstrating a clear distinction between conditions. There are 42 missing values, accounting for 7.5% of the dataset.

**Supplementary Table 1. Hyperparameter Tuning Results.** The optimization results aimed at minimizing RMSE were obtained by evaluating different combinations of feature selection methods, regression models and their respected hyperparameters.

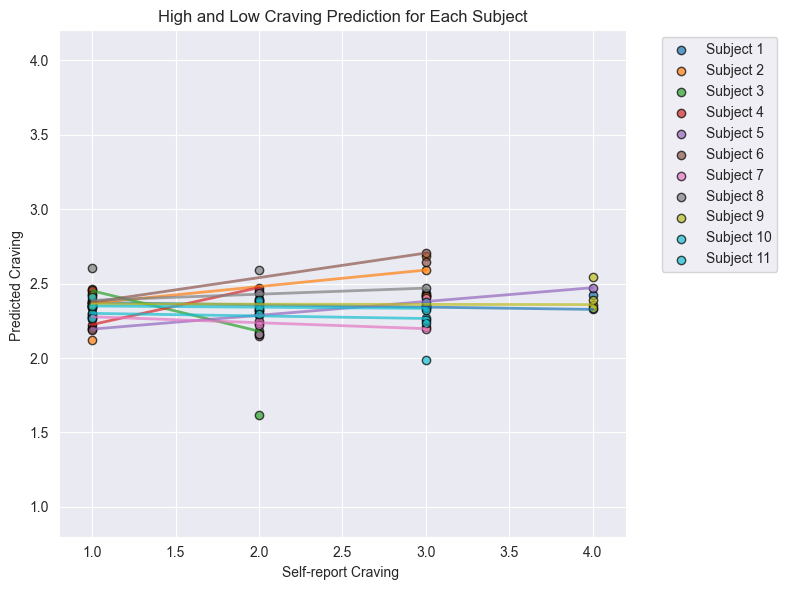


**Supplementary Figure 3. Out-of-sample results for PCA (n=100) and linear regression.** The lines connect the points representing the lowest craving on the x-axis and its predicted value on the y-axis to the points representing the highest craving on the x-axis and its predicted value on the y-axis for each subject in the out-of-sample set.
